# Determining the Mutation Bias of Favipiravir in Influenza Virus Using Next-Generation Sequencing

**DOI:** 10.1128/JVI.01217-18

**Published:** 2019-01-04

**Authors:** Daniel H. Goldhill, Pinky Langat, Hongyao Xie, Monica Galiano, Shahjahan Miah, Paul Kellam, Maria Zambon, Angie Lackenby, Wendy S. Barclay

**Affiliations:** a Public Health England, London, United Kingdom; b Department of Virology, Faculty of Medicine, Imperial College, London, United Kingdom; Icahn School of Medicine at Mount Sinai

**Keywords:** favipiravir, mutation bias, next-generation sequencing, Primer ID, influenza

## Abstract

Favipiravir is a broad-spectrum antiviral drug that may be used to treat influenza. Previous research has identified that favipiravir likely acts as a mutagen, but the precise mutation bias that favipiravir induces in influenza virus RNAs has not been described. Here, we use next-generation sequencing (NGS) with barcoding of individual RNA molecules to accurately and quantitatively detect favipiravir-induced mutations and to sample orders of magnitude more mutations than would be possible through Sanger sequencing. We demonstrate that favipiravir causes mutations and show that favipiravir primarily acts as a guanine analogue and secondarily as an adenine analogue resulting in the accumulation of transition mutations. We also use a standard NGS pipeline to show that the mutagenic effect of favipiravir can be measured by whole-genome sequencing of virus.

**IMPORTANCE** New antiviral drugs are needed as a first line of defense in the event of a novel influenza pandemic. Favipiravir is a broad-spectrum antiviral which is effective against influenza. The exact mechanism of how favipiravir works to inhibit influenza is still unclear. We used next-generation sequencing (NGS) to demonstrate that favipiravir causes mutations in influenza RNA. The greater depth of NGS sequence information over traditional sequencing methods allowed us to precisely determine the bias of particular mutations caused by favipiravir. NGS can also be used in a standard diagnostic pipeline to show that favipiravir is acting on the virus by revealing the mutation bias pattern typical to the drug. Our work will aid in testing whether viruses are resistant to favipiravir and may help demonstrate the effect of favipiravir on viruses in a clinical setting. This will be important if favipiravir is used during a future influenza pandemic.

## Abstract (precis)

New antiviral drugs are needed as a first line of defense in the event of a novel influenza pandemic. Favipiravir is a broad-spectrum antiviral which is effective against influenza. The exact mechanism of how favipiravir works to inhibit influenza is still unclear. We used next-generation sequencing (NGS) to demonstrate that favipiravir causes mutations in influenza RNA. The greater depth of NGS sequence information over traditional sequencing methods allowed us to precisely determine the bias of particular mutations caused by favipiravir. NGS can also be used in a standard diagnostic pipeline to show that favipiravir is acting on the virus by revealing the mutation bias pattern typical to the drug. Our work will aid in testing whether viruses are resistant to favipiravir and may help demonstrate the effect of favipiravir on viruses in a clinical setting. This will be important if favipiravir is used during a future influenza pandemic.

## INTRODUCTION

Influenza virus is responsible for the deaths of between 290,000 to 650,000 people globally each year (1). The emergence of a novel strain of influenza in humans could lead to an influenza pandemic with significant mortality worldwide (2). While vaccination provides good levels of protection against seasonal influenza, at the start of a pandemic, antiviral drugs would be the frontline of defense during a period of development of a specific vaccine (3). Historically, there have been only two licensed classes of antiviral drug for influenza: adamantanes and neuraminidase inhibitors (NAIs). Adamantanes are no longer in clinical use as almost all circulating viruses are resistant (4, 5). Furthermore, some previous seasonal viruses have shown high levels of resistance to the most commonly administered NAI, oseltamivir (6), and oseltamivir-resistant A(H7N9) viruses with pandemic potential have emerged and are transmissible between ferrets (7–9). New drugs are needed for treatment of seasonal influenza as well as for pandemic preparedness and a number of drug classes are under development, including compounds that target the viral RNA-dependent RNA polymerase (RdRp) (10). In 2014, favipiravir, an antiviral drug developed by Toyama, was licensed for use in Japan against emerging influenza viruses that exhibit resistance to other antivirals (11). However, the exact mechanism through which favipiravir exerts an antiviral effect on influenza is unclear. An increased knowledge of the mechanism of action of favipiravir could be useful in determining whether specific viruses are less susceptible and evaluating the potential for the emergence and transmission of resistant viruses.

Favipiravir is a nucleoside analogue that is active against all subtypes of influenza and has shown a potent antiviral effect both *in vitro* and *in vivo* (12–17). Favipiravir has completed a phase III clinical trial in Japan and has undergone a phase III trial in the United States (18). Favipiravir has also been shown to be active *in vitro* and in animal models against a wide range of RNA viruses, some for which there are no licensed drugs as a treatment option (18–25). There is strong evidence that favipiravir acts as a mutagen by incorporating into both positive and negative stranded RNA and being aberrantly copied as multiple bases (15, 26–30). This is thought to be a different mechanism of action from ribavirin, another broadly acting nucleoside analogue that has been used previously to treat influenza (26, 31). Studies have shown that favipiravir competes against guanine and adenine to be incorporated into RNA and is noncompetitive against cytosine and uracil (30, 32–34). This would suggest that favipiravir acts as a purine analogue and should cause mostly transition mutations. Studies measuring the mutation bias of favipiravir in influenza have had mixed results. Baranovich et al. used Sanger sequencing of virus passaged in the presence of drug to show a C→U and a G→A mutation bias, as expected, but also saw a G→U mutation bias after 48 h of exposure to favipiravir (27). Vanderlinden et al. also used Sanger sequencing to show a C→U and G→A bias after a passaging experiment and showed an increase in Shannon entropy using next-generation sequencing (NGS) (31). However, in contrast to studies using Sanger sequencing, Marathe et al. reported a slight bias toward transversions in influenza virus-infected mice treated with favipiravir using NGS (35). Studies with other viruses have demonstrated mutation patterns that suggest that favipiravir acts as a purine analogue (28, 29, 36, 37). Interestingly, several studies with favipiravir and influenza have suggested that favipiravir acts not as a mutagen but as a chain terminator preventing the extension of the RNA strand after incorporation (32, 33). A primer extension study suggested that the block could occur with a single molecule of favipiravir (32), but other studies have suggested that chain termination occurs following the incorporation of two molecules of favipiravir (30, 33, 34).

In this study, we used NGS to determine the mutation bias of favipiravir on influenza virus RNAs. We used two methods of analysis: the first method uses Primer ID, which is a technique for labeling each individual cDNA molecule with a barcode during reverse transcription to account for PCR and sequencing errors but not reverse transcription errors (38–40). This technique can very precisely uncover the mutation bias by analyzing small, targeted areas of the genome. Primer ID has been used on influenza but never to measure mutation bias (40–42). The second method developed a novel analysis of data obtained from a standard sequencing pipeline as would be found in many National Influenza Centres or public health laboratories. This showed the mutation bias induced by drug treatment over the whole genome was similar to that detected using the precise Primer ID methodology and confirmed that the effect of favipiravir could be readily measured using NGS from a standard sequencing pipeline.

(This article was submitted to an online preprint archive [43].)

## RESULTS

### Primer ID allows calculation of mutation bias and relative mutation rate.

**FIG 1:**
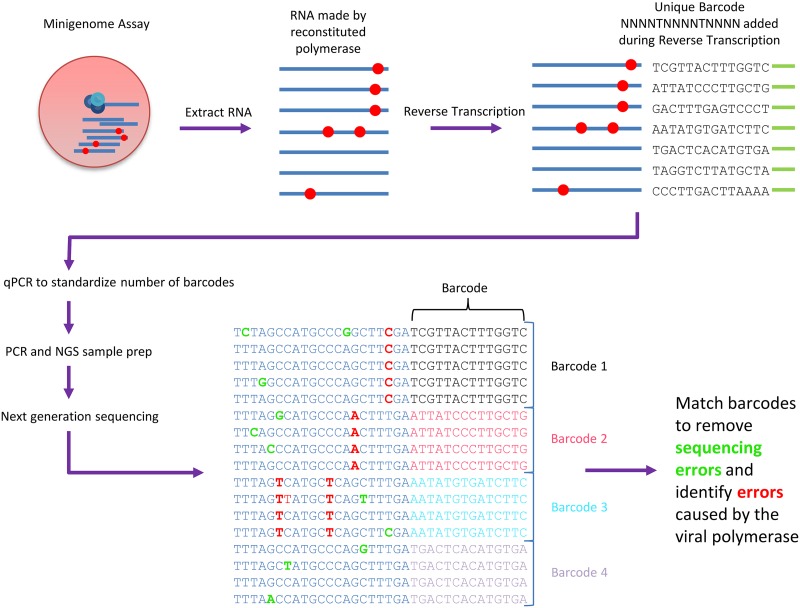
Primer ID method for determining mutation bias. RNA was extracted, and a unique barcode of the form NNNNTNNNNTNNNN was added during reverse transcription. qPCR was used to standardize the number of barcodes for NGS. Samples were sequenced, and barcodes were matched to allow the removal of PCR and sequencing errors.

In order to determine the mutagenic effect of favipiravir, we used NGS with Primer ID to analyze the products of a minigenome assay (38), which allowed for the unbiased measurement of mutations (Fig. 1). When sequencing virus, particularly over several rounds of replication, a proportion of possible mutations will not be measured, since they would cause too large a fitness cost to the virus and thus will not be amplified. To avoid this scenario, we sequenced the reporter gene from the minigenome assay since the reporter protein has no effect on further RNA accumulation. Thus, this strategy should reveal the complete spectrum of mutations caused by replication in the presence of favipiravir. Primer ID is a method which labels each cDNA molecule with a unique barcode during reverse transcription (Fig. 1). This method allowed us to examine a large number of independent mutational events, as each mutation could be associated with an individual cDNA molecule. In addition, by comparing multiple sequencing reads with the same barcode, we could remove sequencing errors since these would not appear in the majority of the reads. The sample without favipiravir provides a baseline mutation rate consisting of the background mutation rate of the influenza virus polymerase plus mutations caused by the reverse transcriptase during reverse transcription. Drug-treated samples can be compared to this sample to measure how favipiravir increased the mutation rate.

**FIG 2:**
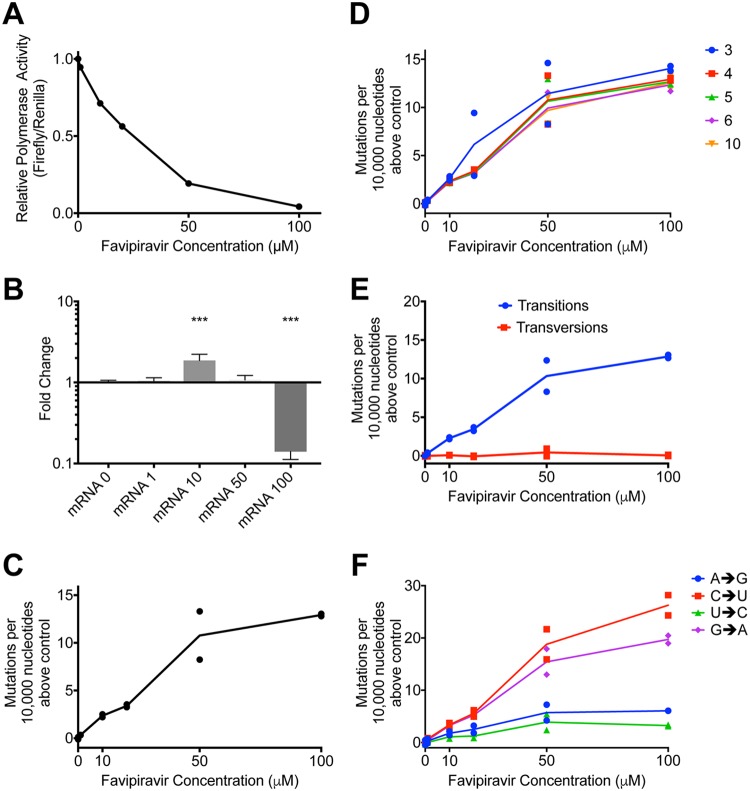
Favipiravir causes transition mutations which reduces polymerase activity in a minigenome assay. (A) Minigenome assay. Plasmids were transfected into 293T cells, and favipiravir was added. At ∼21 h, the cells were lysed, and the luciferase activity was measured. The relative polymerase activity is calculated as the firefly activity/*Renilla* activity. (B) qPCR was performed on the luciferase reporter mRNA from a minigenome assay. ΔΔ*C_T_* was calculated using 18S RNA, and results are shown normalized to the drug-free control (*n* = 6; ***, *P* < 0.001). (C) A reporter plasmid (HA pol1) from the minigenome above was sequenced using Primer ID and NGS. The mutations were tallied as described in Materials and Methods. Two independent biological samples from separate wells of the minigenome assay were sequenced for each concentration of drug in the same sequencing reaction. The numbers of mutations per 10,000 nucleotides above the average of the two control samples were compared for each sample. Baseline = 0.8 mutations per 10,000 nucleotides. (D) The cutoff for the number of reads necessary to include a barcode was systematically varied, and the number of mutations per 10,000 nucleotides above the control was plotted for each sample. Cutoffs of 3, 4, 5, 6, and 10 gave baselines of 1.3, 0.8. 0.7, 0.7, and 0.7 mutations per 10,000 nucleotides, respectively. (E) The number of mutations per 10,000 nucleotides above the control for each sample was calculated for transitions and transversions (baseline transitions = 0.6 mutations per 10,000 nucleotides; baseline transversions = 0.3). (F) The number of mutations per 10,000 nucleotides above the control for each sample was calculated for each class of transition mutation. The values were calculated as the mutation rate for an individual base (baseline A→G = 0.7 mutations per 10,000 nucleotides; C→U = 0.4; G→A = 0.3; U→C = 0.8).

We reconstituted influenza RdRp *in situ* by expressing the polymerase proteins and nucleoprotein from transfected plasmids. We introduced two virus-like RNA templates, one in which the authentic open reading frame was replaced by the firefly luciferase gene and one that represented RNA segment 4 and encoded H3 hemagglutinin (HA). The transfected cells were incubated in the presence of favipiravir. Increasing concentrations of favipiravir from 1 to 100 μM caused a reduction in the activity of the luciferase reporter (Fig. 2A). However, quantitative reverse transcription-PCR analysis of the amount of H3 HA mRNA accumulated revealed no decrease in mRNA levels that would account for the loss of luciferase activity at least up to 50 μM drug (Fig. 2B). At 100 μM favipiravir, there was a significant reduction in mRNA (*P* < 0.0001). This suggested that at doses up to 50 μM, the inhibitory effect of favipiravir in the minigenome assay was mostly caused by mutagenesis and not through chain termination, which could have played a role at the highest dose of drug.

In order to test how favipiravir affected the mutation rate of the reconstituted viral polymerase, we sequenced the positive-stranded H3 HA RNAs. Since each individual barcode represents a single cDNA molecule and therefore a single RNA molecule, we calculated consensus sequences for each barcode. Mutations which did not appear in a majority of reads were ascribed to PCR or sequencing error and removed from further analyses. In total, we analyzed 6,623 substitutions in ∼6,900,000 bases of sequencing data. Figure 2C shows the number of mutations per 10,000 nucleotides above the baseline (0 μM favipiravir) for each sample. As the concentration of favipiravir increased, the number of mutations increased. At the highest concentration of favipiravir tested (100 μM), there would be an additional 13 errors per 10,000 nucleotides on average compared to the control. We varied the cutoff for the number of sequencing reads needed to include a barcode (Fig. 2D). The choice of cutoff did not significantly alter the results for values of <10 reads. We chose a cutoff of four reads per barcode since this removed some errors associated with low numbers of reads per barcode while including the majority of the data.

We next categorized the mutations identified by sequencing as transitions or transversions or as the individual base-pair mutations (Fig. 2E and F). Our results confirmed that the main cause of the increase in mutation rate was transition mutations (Fig. 2E). There was no increase in the rate of transversion mutations as the concentration of favipiravir increased (F-test, F = 0.4593, df = 1,4, *P* = 0.5351). Figure 2F shows the increase in the likelihood of different categories of mutations compared to the control. The most common transitions were C→U and G→A mutations that would be induced when favipiravir is acting as a guanine analogue. However, there was also a smaller increase in the reverse transitions from U→C and A→G where favipiravir acts as an adenine analogue. On average, there was an ∼3.5-fold increase in the rate of C→U or G→A mutations compared to U→C or A→G mutations.

### Primer ID sequencing of viruses confirms that favipiravir causes mutations.

**FIG 3:**
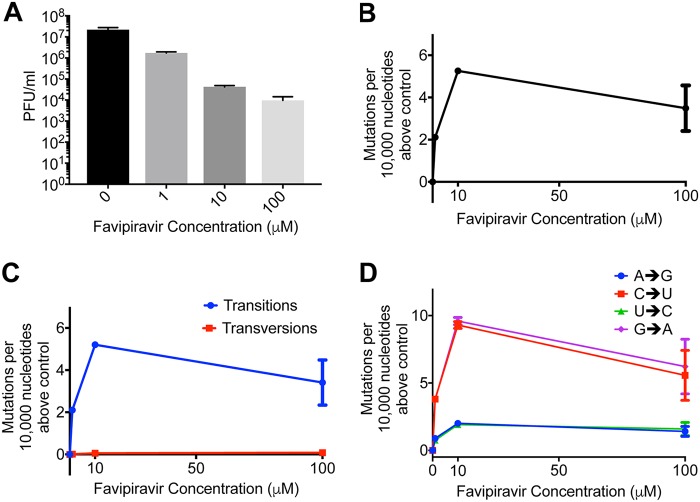
Favipiravir causes transition mutations reducing viral fitness. (A) Virus was added to MDCK cells at a high MOI of 1.3, and favipiravir was added at an appropriate concentration diluted in DMSO. The supernatant was titered by plaque assay after 20 h, and the titer was calculated in PFU/ml (*n* = 3). (B) After 18 h, the cells were lysed, and the RNA was extracted for sequencing using Primer ID. The number of mutations per 10,000 nucleotides above the control was plotted for each sample. The baseline mutation rate was 0.8 mutations per 10,000 nucleotides (*n* = 3). (C) The number of mutations per 10,000 nucleotides above the control for each sample was calculated for transitions and transversions (baseline transitions = 0.5 mutations per 10,000 nucleotides; baseline transversions = 0.3). (D) The number of mutations per 10,000 nucleotides above the control for each sample was calculated for each class of transition mutation (baseline A→G = 0.6 mutations per 10,000 nucleotides; C→U = 0.3; G→A = 0.6; U→C = 0.4). The values were calculated as the mutation rate for an individual base.

We next tested whether we could use Primer ID to measure the increase in mutation rate of RNAs generated during virus infection. To minimize the loss of viral RNAs that contained mutations rendering the virus nonviable, we infected cells at a high multiplicity of infection (MOI) so that there was only a single replication cycle. We first confirmed that favipiravir inhibited influenza under these conditions (Fig. 3A). There was a >1,000-fold reduction in infectious titer of influenza A/Eng195/2009 A(H1N1)pdm09 virus (Eng 195) after 24 h infection at high concentrations of favipiravir and a 10-fold reduction at a 1 μM concentration of the drug. We extracted RNA from the cells and sequenced the vRNA of RNA segment 2 with appropriate barcoded primers. In total, we analyzed ∼56,000,000 bases and found 25,441 substitutions. All concentrations of favipiravir showed an increase in mutation rate compared to the no drug control (Fig. 3B). The mutation rate caused by favipiravir was ∼3-fold higher at 10 μM than at 1 μM, but surprisingly, the mutation rate at 100 μM favipiravir was lower than at 10 μM. The increase in mutation rate at all concentrations of favipiravir was almost entirely due to transitions (Fig. 3C). The mutation bias measured was similar to that seen using the minigenome assay with C→U and G→A mutations occurring most frequently (Fig. 3D).

### NGS can reveal mutation bias.

**FIG 4:**
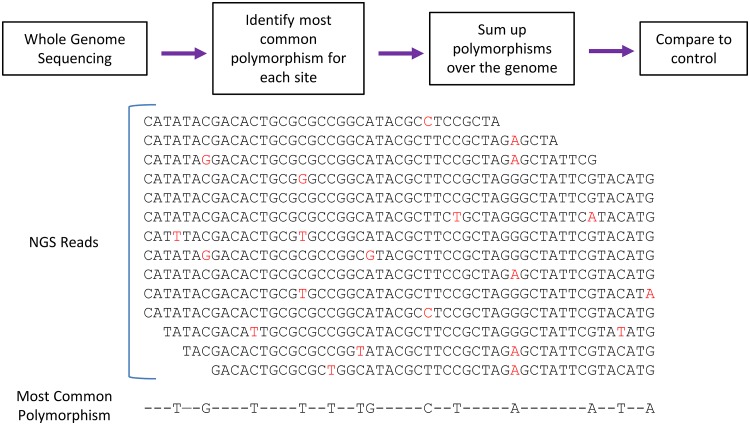
Method to analyze mutation bias from whole-genome NGS data. Whole-genome sequencing data from a standard sequencing pipeline were aligned to a reference. The most common polymorphism for each site in the genome was calculated. These polymorphisms were summed up, and the mutation bias of different samples can be compared.

**FIG 5:**
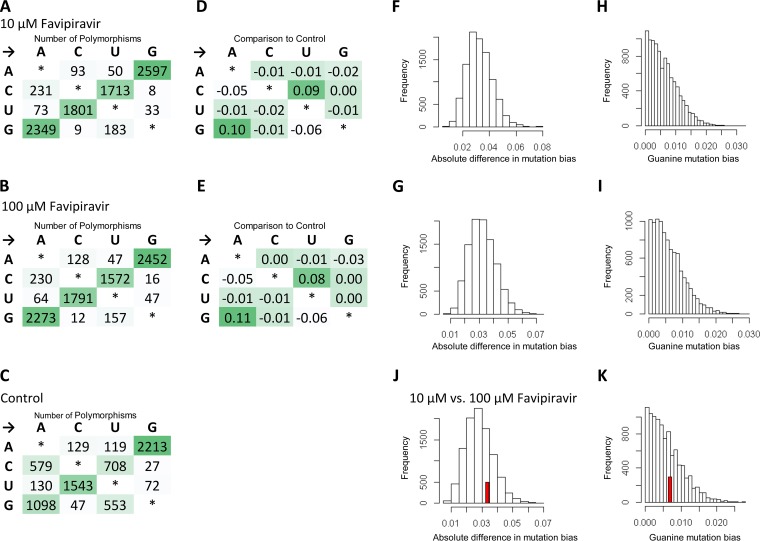
NGS data show that favipiravir acts as a guanine analogue. Virus was added to MDCK cells at a high MOI of 1, and drug was added as previously described. Supernatant was taken, sequenced, and analyzed as described in Materials and Methods. The most common polymorphism for each base is shown for virus exposed to 10 μM favipiravir (A), 100 μM favipiravir (B), and a drug-free control (C). (D and E) A comparison to the control shows the difference in percentage for each class of mutations, revealing a mutation bias for 10 μM favipiravir (D) and 100 μM favipiravir (E). A permutation analysis was performed on the mutation data. The substitutions were randomized between the treatment and control, and either the total absolute difference in mutation bias was calculated (F, G, and J) or the bias for acting as a guanine analogue was calculated (H, I, and K). A total of 10,000 permutations were performed for each analysis. The red bars show the observed value where it occurs within the values generated by the permutations. (F) The mutation bias for 10 μM favipiravir was compared to the control (observed value = 0.39; *P* < 10^–4^). (G) The mutation bias for 100 μM favipiravir was compared to the control (observed value = 0.37; *P* < 10^–4^). (H) The difference in bias for guanine mutations for 10 μM favipiravir compared to the control (observed value = 0.19; *P* < 10^–4^). (I) The difference in bias for guanine mutations for 100 μM favipiravir compared to the control (observed value = 0.19; *P* < 10^–4^). (J) The mutation bias for 10 μM favipiravir was compared to 100 μM favipiravir (observed value = 0.03; *P* = 0.26). (K) The difference in bias for guanine mutations (observed value = 0.007; *P* = 0.34). *n* = 1.

**FIG 6:**
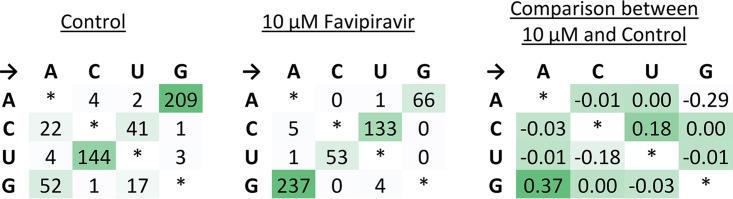
The data from Fig. 5 were reanalyzed for 10 μM favipiravir and the control sample using only the 500 sites with the largest degree of polymorphism.

The experiments with Primer ID showed the mutation rate and bias for a small targeted portion of influenza genome. Next, we wanted to test whether we could measure the mutagenic effect of favipiravir using a standard NGS pipeline typical of those in public health laboratories (Fig. 4). Eng195 virus was propagated at a high MOI for 24 h in the presence of 10 or 100 μM favipiravir. The supernatant was titered by plaque assay to confirm that favipiravir had an inhibitory effect on the virus, and there was >2-log inhibition at 10 μM and >4-log inhibition at 100 μM. We extracted RNA from virus particles in the supernatant and used NGS to obtain sequence data from the population of surviving viruses. In order to analyze mutation bias using next-generation data, it is necessary to ensure that the mutations used for the analysis are independent so that the same mutation occurring on multiple reads is not counted as multiple mutational events but as a single mutational event. Therefore, we treated each base in the influenza genome independently and recorded only the most common mutation (if any) for each site (Fig. 4). Taking these sites in aggregate will give a combination of true mutations, as well as other sources of error, most notably sequencing error. Figure 5 shows the sum of mutations over the whole genome for viruses propagated in 10 μM (Fig. 5A) or 100 μM favipiravir (Fig. 5B) or for control viruses which were not exposed to favipiravir (Fig. 5C). Comparing the pattern of mutations between the control viruses and the viruses exposed to drug allowed us to control for sequencing errors (Fig. 5D and E). The pattern of mutations seen in both samples exposed to favipiravir were significantly different to the control (permutation analysis, *P* < 10^–4^; Fig. 5F and G). The mutation bias was caused by an excess of C→U and G→A transitions compared to control viruses (permutation analysis, *P* < 10^–4^, Fig. 5H and I). There was no significant difference between the mutation bias at the two different concentrations of favipiravir tested (permutation analysis, *P* = 0.26, Fig. 5J and K). To demonstrate further that this method measures a true mutational signal, we took the 500 sites with the highest degree of polymorphism and repeated the analysis (Fig. 6). The new analysis showed an increased effect size strongly suggesting that mutations caused by favipiravir lead to a signal in the sequencing data that is not masked by sequencing error. We chose to use the relative proportion of the mutation types to compare between samples, as opposed to the absolute number of polymorphisms. This was a conservative choice since there may be biases between samples that could affect the absolute numbers of polymorphisms due to the number of viruses in the sample.

## DISCUSSION

**FIG 7:**
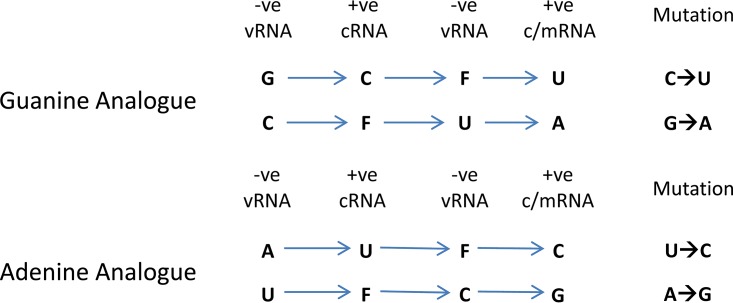
Schematic showing how favipiravir causes mutations during positive- and negative-strand synthesis. F, favipiravir.

In this study, we used two different methods of analyzing next-generation sequencing data in order to show that favipiravir acts as a mutagen with a distinct bias to induce transitions in influenza virus RNAs. The first method used Primer ID to measure precisely the increase in mutation rate and the mutation bias of the influenza polymerase caused by favipiravir in an *in vitro* system. We confirmed that favipiravir has a bias for transition mutations and acts as a purine analogue (17, 26, 32, 33). We were able to demonstrate that favipiravir competed primarily with guanine and secondarily with adenine, resulting in an increase in C→U and G→A mutations at higher concentrations of drug and a lower rate of increase in U→C and A→G mutations (Fig. 7). The second method used data from whole-genome sequencing of viruses that had been exposed to favipiravir during single cycle replication and showed that viral populations exposed to favipiravir had a distinct bias for transition mutations, specifically C→U and G→A mutations.

Previous methods of sequence analysis for determining mutation bias in influenza RNAs induced by favipiravir have relied on Sanger sequencing of individual viral clones (27, 31). This technique is laborious and results in the detection of relatively few mutations: on the order of 100 mutations for an entire experiment (27, 31). Furthermore, the technique can be biased due to selection of beneficial mutations which may appear in multiple clones or to accidentally counting an initial polymorphism in the population as a mutational event that occurred in multiple clones. Sequencing a small region of the genome across many clones is especially prone to this error. NGS with Primer ID is a powerful technique which allowed us to examine orders of magnitude more mutations than Sanger sequencing and was less prone to biases present in examining a small number of mutations. Primer ID allowed us to remove sequencing error from NGS data and to detect changes in mutation rate and mutation bias (38, 39). Primer ID identified thousands of mutations in a single sample exposed to favipiravir, a number which would be impractical using Sanger sequencing. We were able to show that favipiravir acts as both a guanine and an adenine analogue, whereas Sanger sequencing was not sensitive enough to measure the lower rate of adenine mutations (27).

The use of the minigenome assay allowed us to see all mutations generated by polymerase and not just those that would allow viable viruses. Pauly et al. have recently shown that the mutation rate for influenza has been significantly underestimated by only counting mutations which occur in plaque-forming viruses (44). Sequencing only viruses which have exited the cell ignores mutations that cause defects in packaging or cellular exit. In contrast, since the mRNA from the reporter in the minigenome assay is not translated to a protein that can impact viral fitness, the full spectrum of drug-induced mutations can be seen. Allowing for multiple rounds of virus replication makes it difficult to see strongly deleterious mutations, which make up a significant proportion of the mutations for influenza, because they are selected against (45). The minigenome assay has no selection on mutations and does not suffer from this bias. However, when we used a Primer ID approach to sequence a small portion of the viral genome from PB1 amplified during virus infection rather than in the minigenome assay, we found, in contrast to the minigenome sequencing, that there was no increase in the mutation rate at the highest concentrations of favipiravir. This is likely due to selection against deleterious mutations that occurs even in a single cycle of replication. Favipiravir causes mutations randomly, and therefore there will be a distribution in the number of mutations during each strand replication. Some RNAs will have many mutations, whereas others will have fewer. The majority of the RNA that was sequenced will come from viruses that have suffered few mutations, since viral RNAs with more mutations will interfere with ongoing replication. Therefore, the more successful favipiravir is at causing mutations, the greater the bias to sequencing the small number of viruses with fewer mutations. This most likely explains why the mutation rate we measured appeared lower at 100 μM favipiravir than at 10 μM.

Although Primer ID can remove sequencing error, it is still impossible to distinguish between errors due to the flu polymerase and the reverse transcriptase used during the Primer ID reaction. A recent study has suggested that care must be taken since these two error rates are the same order of magnitude (44). For this reason, we have not reported an absolute error rate but a relative error rate compared to the drug-free baseline sample. However, for our experiments, the mutation rate caused by favipiravir was much higher than the calculated baseline mutation rate caused by reverse transcription errors plus errors naturally caused by the influenza polymerase. Furthermore, as all samples underwent identical processing, there is no reason to believe that the error rate during reverse transcription differed between samples, and this is therefore unlikely to bias our data. Care should be taken before comparing samples that have not been prepared concurrently, especially if different reverse transcription enzymes are used.

One disadvantage to Primer ID is that it sequences only a small part of the genome. This potentially could lead to mutation biases if that part of the genome was under strong selection or due to local sequence structure. Because we sampled only one region of the HA, we could not test whether there were specific structural differences between the HA sequence and other flu segments leading to mutational hot spots. One possibility would be to use multiple sets of barcoded primers to sequence a larger area (42). However, the similarity between our analysis of RNAs from Primer ID versus whole-genome sequencing suggests we did not inadvertently sample a mutational hot spot. The precision and ease with which Primer ID was able to distinguish mutation bias and observe changes in mutation rate leads us to suggest that it could become a standard method for analyzing the effects of nucleoside analogues and other mutagenic drugs.

Our second method of analysis sequenced the whole influenza genome in populations of viruses that had been exposed to favipiravir and a control population that was not exposed to the drug, as might be found in a clinical setting. The main disadvantage of this technique is that it is unable to distinguish between sequencing error and “true” errors caused by the flu polymerase. Therefore, it is not possible to quantify the actual number of errors due to polymerase nor was the method sensitive enough to demonstrate any increase in the rate of U→C and A→G mutations. Despite these limitations, there are several advantages to this method that may prove to be of use in clinical settings. This method is extremely simple to use since the viruses can be entered into the standard influenza sequencing pipeline without any additional processing steps and could also be used to reanalyze data that had been previously collected. The analysis also encompasses the whole genome and so is resistant to any biases caused by local RNA structure, nor is it biased by single polymorphisms that may have been present in the initial populations. If favipiravir is used in a clinical setting, this method may be a simple way to show that favipiravir is having a measurable effect by comparing viral mutations in pretreatment and posttreatment samples.

In contrast to our finding that favipiravir acts as a purine analogue, a previous study that used NGS to determine the mutation bias of favipiravir *in vivo* found an excess of transversion mutations (35). The analysis in Marathe et al. counted each individual NGS read as a separate mutational event, which may have led to a bias, since mutations from preexisting polymorphisms or mutations that are positively selected will be counted multiple times. In contrast, our method of analyzing NGS data ensured that mutations were independent by only counting one mutation at each site in the genome (Fig. 4). Many recent papers that analyze NGS data use a cutoff, e.g., 5 or 1% of reads below which variants are not counted (31, 35, 37). However, using a cutoff discards a large amount of the sequence data since only a small proportion of the sites are included. Our analysis (Fig. 5) used all of the sequencing data without imposing a cutoff, and this led to increased noise in the data but ensured that there was no bias toward preexisting polymorphisms or variations in sequencing depth. We also tested the mutational bias by only counting the 500 sites with the largest degree of polymorphism (Fig. 6), which showed results similar to those from our main analysis though potentially with less noise. This suggests that imposing a cutoff on variants will not bias the results if the sequencing contains enough variants that positive selection and preexisting polymorphisms are unlikely to influence the results.

Our data showed that favipiravir acts as a mutagen with a bias toward transitions, in agreement with most other studies of this drug’s effect on RNA viruses (27, 28, 31). We found in the minigenome assay that at lower concentrations of favipiravir, there was no evidence suggesting that the drug was acting as a chain terminator since there was no reduction in the amount of mRNA despite a reduction in reporter gene activity (Fig. 2A and B). At the highest concentration tested (100 μM), there was a reduction in mRNA which could have been caused by chain termination or through introduced mutations preventing RNA replication. Although we demonstrated that favipiravir acted as a mutagen on virus (Fig. 3), we did not exclude the possibility that it could also be acting as a chain terminator. Biochemically, favipiravir acts as a purine analogue binding to either C or U in place of G or A, respectively. The most common mutations caused by favipiravir were C→U and G→A. These mutations were caused by favipiravir binding to C in place of a G on the positive- or negative-strand synthesis and subsequently pairing with a U in the next synthesis cycle (Fig. 7). The reverse transitions caused by favipiravir binding to U happened at an ∼3.5-fold-lower rate. This confirms that favipiravir is most competitive against G, as had been previously seen in primer extension assays (32, 33).

NGS is a powerful technique for analyzing mutational data and determining mutational biases. Care must be taken to perform analyses which minimize potential biases by ensuring that mutations are only counted when they occur independently of each other. We used NGS to show that favipiravir is acting as a mutagen causing multiple additional mutations per influenza genome on average at higher concentrations of favipiravir. Lethal mutagenesis of influenza virus is a viable antiviral strategy and may be difficult to evolve resistance against clinically (46). Our increased knowledge of the precise mechanism of favipiravir means that we are better placed to test whether the drug is having a clinical effect, as well as to see whether viruses are becoming resistant to favipiravir. This will be important when this drug is used in a pandemic situation.

## MATERIALS AND METHODS

### Reagents, cells and viruses.

Favipiravir, kindly provided by Toyama Chemical Company under a material transfer agreement, was reconstituted in dimethyl sulfoxide (DMSO) and frozen into aliquots. MDCK and 293T cells were grown in Dulbecco modified Eagle medium (Gibco) with the addition of 10% fetal bovine serum (Labtech), 1% nonessential amino acids (Gibco), and 1% penicillin-streptomycin (Sigma-Aldrich). A/England/195/2009 (Eng195) is an early isolate from the 2009 A(H1N1) pandemic provided by Public Health England (PHE).

### Minigenome assay.

Four pCAGGS plasmids encoding the polymerase (PA, PB1, and PB2) and NP from influenza A/England/195/2009 A(H1N1)pdm09 virus were transfected using Lipofectamine 3000 (Invitrogen) into 293T cells in 24-well plates. In addition, we transfected plasmids directing expression from a polymerase (Pol) I promoter of either a firefly luciferase gene in negative sense flanked with influenza A noncoding sequence from the NS segment or the HA gene segment from influenza A/Victoria/3/75 H3N2 virus (Vic75), and a Pol II *Renilla* luciferase plasmid as a transfection control. Cells were lysed with 200 μl of passive lysis buffer (Promega), and the polymerase activity was measured using a dual-luciferase reporter assay (Promega) on a FLUOstar Omega plate reader (BMG Labtech). Pol activity is reported as firefly luciferase activity normalized by *Renilla* activity.

### NGS with primer ID.

At 24 h after transfection, 293T cells from the minigenome assay were lysed, and RNA was extracted using the RNA minikit (Qiagen). The reverse transcription primer for primer ID (5ʹ-TGCGTTGATACCACTGCTTTNNNNTNNNNTNNNNCCCAGTCCAAGTGAAACCCTC-3ʹ) consisted of a PCR tag, a random barcode of the form NNNNTNNNNTNNNN, and a sequence specific to the H3 HA. Reverse transcription was performed with Superscript III (Thermo Fisher). qPCR using SYBR green (Thermo Fisher) was used to calculate the number of cDNA molecules to use for each PCR. A total of 20,000 to 40,000 molecules were used for each reaction. The PCR primers were 5ʹ-CGGGGAAAATATGCAACAATCCT-3ʹ and 5ʹ-TGCGTTGATACCACTGCTTT-3ʹ. The PCR product was designed to be 279 bases to avoid any fragmentation step during sample preparation, ensuring the barcode was not sheared from the sample. Sample preparation was performed using NEBNext Ultra kit (NEB). Samples were sequenced giving 150-bp paired end reads on an Illumina MiSeq. Sequencing data for the samples were processed and analyzed using custom scripts in Python and R. Reads were first paired to form a single sequence and subjected to quality control using QUASR v7.01 (47) to retain reads with a median phred score of 20 and minimum read length of 250 bp. Intact barcode sequences were extracted from the read pairs; any sequences without a fully formed barcode or with errors in the internal Ts of the barcode were discarded. Consensus sequences were generated for each barcode that had more than three reads with the consensus taken as the majority of the reads. Samples for which there was no majority read were discarded, since this could be an example of two RNA sequences having the same barcode (48). The consensus sequences were mapped and compared to the Vic75 reference and any variants were extracted. We subsequently decided to use a more stringent cutoff four reads per barcode to minimize errors caused by barcodes with a low number of reads. We present all our sequencing results as mutations in positive orientation, as would have been seen in the mRNA. All of the the sequencing data in this study are archived at https://www.ebi.ac.uk/ena under project PRJEB28478. The code that was used to run these sequence analyses can be found at https://github.com/Flu1.

### qPCR.

RNA was extracted from a minigenome assay. Specific primers were used to reverse transcribe mRNA from the firefly luciferase, as previously described (49). qPCR was performed with SYBR Green using 18S RNA as a control. ΔΔ*C_T_* was calculated, and the results are shown normalized to the drug-free control.

### Next-generation viral sequencing with Primer ID.

A total of 1.2 × 10^6^ cells were inoculated with Eng195 at an MOI of 1.5, followed by incubation at 37°C for 18 h in serum-free medium with added 1 μg/ml trypsin (Worthington) and with different concentrations of favipiravir diluted in DMSO. Control wells contained DMSO without favipiravir. After 18 h, samples were taken from the supernatant and plaque assayed on MDCK cells to determine final viral titer. RNA was extracted from the cells using an RNeasy kit (Qiagen). Sequencing was performed as described above, except that the Primer ID RT primer contained sequence specific for PB1 vRNA (5ʹ-TGTCCAGCACGCTTCAGGCTNNNNTNNNNTNNNNAGAAGATGGTCACGCAAAGAA-3ʹ) and the PCR product was 302 bases long, including the PCR primers 5ʹ-TCACAACATTTGCCAGTTTGG-3ʹ and 5ʹ-TGTCCAGCACGCTTCAGGCT-3ʹ. On analyzing the sequencing data, a site which varied considerably in all samples was detected that was likely a polymorphism in the initial population. This site was removed from all analyses.

### NGS without Primer ID.

A total of 1.2 × 10^6^ cells were inoculated with England 195 at an MOI of 1, followed by incubation at 37°C for 24 h as described above. Control wells contained DMSO but no favipiravir. After 24 h, samples were taken from the media, and titers were determined on MDCK cells by plaque assay. Whole-genome NGS was performed using a pipeline at PHE. RNA was extracted from viral lysate using easyMAG (bioMérieux). One-step RT-PCR was performed using SuperScript III (Invitrogen), Platinum *Taq* HiFi polymerase (Thermo Fisher), and influenza-specific primers (50). Samples were prepared for NGS using the Nextera library preparation kit (Illumina). Samples were sequenced on an Illumina MiSeq generating a 150-bp paired-end reads. Reads were mapped with BWA v0.7.5 and converted to BAM files using SAMTools (1.1.2). Variants were called using QuasiBAM, an in-house script at PHE. Samples were compared using a permutation analysis to calculate the probability of a magnitude of mutation bias as great as observed given the mutations in the samples. Permutation analyses were performed in R, with 10,000 iterations for each analysis. Mutations were randomized between two samples maintaining the number of mutations found within each sample. The magnitude of the mutation bias was then calculated as the sum of the absolute value of the difference in the relative proportions of each mutation type. The *P* value was then calculated as the number of iterations/10,000 with a value greater than the observed value. A further permutation analysis calculated the probability of a bias of guanine analogue mutations (e.g., C→U and G→A). This analysis was performed as described above but only used the sum of the absolute value of the difference in the relative proportions of C→U and G→A.
